# Exploiting High-Energy Emissions of YAlO_3_:Dy^3+^ for Sensitivity Improvement of Ratiometric Luminescence Thermometry

**DOI:** 10.3390/s22207997

**Published:** 2022-10-20

**Authors:** Jovana Periša, Aleksandar Ćirić, Ivana Zeković, Vesna Đorđević, Milica Sekulić, Željka Antić, Miroslav D. Dramićanin

**Affiliations:** 1 Centre of Excellence for Photoconversion, Vinča Insitute of Nuclear Sciences—National Institute of the Republic of Serbia, University of Belgrade, P.O. Box 522, 11001 Belgrade, Serbia; 2 School of Optoelectronic Engineering, CQUPT-BUL Innovation Institute, Chongqing University of Posts and Telecommunications, Chongqing 400065, China

**Keywords:** luminesce intensity ratio, high-temperature luminescence thermometry, Dy^3+^- activated YAP, third thermalized level

## Abstract

The sensitivity of luminescence thermometry is enhanced at high temperatures when using a three-level luminescence intensity ratio approach with Dy^3+^- activated yttrium aluminum perovskite. This material was synthesized via the Pechini method, and the structure was verified using X-ray diffraction analysis. The average crystallite size was calculated to be around 46 nm. The morphology was examined using scanning electron microscopy, which showed agglomerates composed of densely packed, elongated spherical particles, the majority of which were 80–100 nm in size. The temperature-dependent photoluminescence emission spectra (ex = 353 nm, 300–850 K) included Dy^3+^ emissions in blue (458 nm), blue (483 nm), and violet (430 nm, T 600 K). Luminescence intensity ratio, the most utilized temperature readout method in luminescent thermometry, was used as the testing method: a) using the intensity ratio of Dy^3+^ ions and ^4^I_15/2_→^6^H_15/2/_^4^F_9/2_→^6^H_15/2_ transitions; and b) employing the third, higher energy ^4^G_11/2_ thermalized level, i.e., using the intensity ratio of ^4^G_11/2_→^6^H_15/2/_^4^F_9/2_→^6^H_15/2 _transitions, thereby showing the relative sensitivities of 0.41% K^−1^ and 0.86% K^−1^ at 600 K, respectively. This more than doubles the increase in sensitivity and therefore demonstrates the method’s usability at high temperatures, although the major limitation of the method is the chemical stability of the host material and the temperature at which the temperature quenching commences. Lastly, it must be noted that at 850 K, the emission intensities from the energetically higher levels were still increasing in YAP: Dy^3+^.

## 1. Introduction

Temperature measurements that are based on changes in material luminescence have received a lot of attention in recent years [1,2,3,4,5]. This semi-invasive method offers reliable, precise, fast, and single-point or 2D thermal imaging in a wide temperature range, which can range from cryogenic temperatures to approximately 1700 °C [6]. It can be applied to macroscopic and microscopic systems, large surfaces, in vivo [7,8], fiber-optic probes [9,10,11], corrosive, radioactive environments [12], or high electromagnetic fields.

The luminescence intensity ratio (LIR) of the emission from lanthanide-activated phosphors is the most frequently utilized method in luminescence thermometry. It offers many benefits, such as accuracy and the simplicity of measurements, high reproducibility, and self-referencing. When emissions from adjacent thermalized excited levels are utilized (known as Boltzmann thermometers), the *LIR* can be explained by a Boltzmann distribution [13]:

(1) $$LIR=\frac{I_{H}}{I_{L}}=Be^{-\frac{\Delta E}{kT}},$$

where *I* represents the integrated intensities from higher (*H*) and lower (*L*) energy levels; Δ*E* = *E_H_* − *E_L_* is the energy difference between the excited levels; *B* is the temperature invariant constant (dependent on the properties of the host material); and *k* = 0.695 cm^−1^ K^−1^ is the Boltzmann constant. The performances of temperature-sensitive materials can be assessed by comparing their relative sensitivity, which is a figure of merit that represents the amount of change in the indication with the temperature. The relative sensitivity (S_R_) is presented by [14,15]:

(2) $$S_{R}\left[\%\;K^{-1}\right]=\frac{1}{LIR}\left|\frac{\partial LIR}{\partial T}\right|=\frac{\Delta E}{kT^{2}}$$

It can be seen from Equation (2) that *S_R_* at a specific temperature depends linearly on the energy difference between the thermalized levels. As a rule of thumb, one can consider the energy of 2000 cm^−1^ as the limit above which the thermal energy (at room temperature) is not sufficient for the population of the *H* level [16]. In the case of Dy^3+^ ions, the energy difference between the first two excited levels, ^4^F_9/2_ and ^4^I_15/2_ (approximately 900 cm^−1^), limits the relative sensitivity at room temperature to 1.5% K^−1^. In fact, Boltzmann thermometers show a maximal 2.93% K^−1^ value of relative sensitivity at room temperature in Eu^3+^- activated phosphors with the largest energy difference between the first two excited levels (approximately 1750 cm^−1^) [17]. Yet, to achieve better accuracy of the Boltzmann-type luminescence thermometers, higher values of relative sensitivity are needed since the uncertainty in the measured temperature is inversely proportional to the relative sensitivity. Low sensitivity becomes an even larger problem in measurements at high temperatures due to the relative sensitivity, which is proportional to the square of the inverse temperature (see Equation (1)), i.e., it rapidly decreases with an increase in temperature.

To overcome the above-mentioned limitations of the Boltzmann thermometer and to obtain larger values of relative sensitivity at high temperatures, emissions from higher-energy excited levels can be used in the LIR approach. The thermal energy needed to populate the energetically higher levels is proportional to the temperature, expanding this energy gap limit for the Boltzmann distribution at elevated temperatures. To the best of the authors’ knowledge, there are not many reports on the inclusion of the third thermalized level for the realization of Boltzmann thermometers with emissions from higher energy excited levels, such as: Dy^3+^ activated CaWO_4_ [18] and Lu_1.5_Y_1·5_Al_5_O_12_ [19]; Nd^3+^ activated NaYF_4_ [20]; Er^3+^ activated YF_3_ [21] and Pr^3+^; and Gd^3+^ activated YAl_3_(BO_3_)_4_ [22].

This study aims to investigate the potential of Dy^3+^-activated yttrium aluminum perovskite (YAlO_3_, YAP) for luminescence thermometry at high temperatures using the third thermalized excitation level of Dy^3+^. The Dy^3+^- activated YAP is chosen as a demonstrating system for two reasons: 1) the Dy ion is selected due to the ladder-like structure of energy levels necessary for this approach, and 2) the YAP host is chosen due to its good thermal, mechanical, and optical properties [23,24]. The 2 mol% content of Dy^3+^ was selected according to the study in reference [25]. The Dy^3+^- activated sample was obtained using the modified Pechini method [26], and its usability as a temperature-sensitive material was confirmed by two LIR methods: (a) the traditional method using the intensity ratio of Dy^3+^ ions ^4^I_15/2_→^6^H_15/2_/^4^F_9/2_→^6^H_15/2_ transitions (LIR1), and (b) LIR employing the third, higher-energy ^4^G_11/2_ thermalized level, ^4^G_11/2_→^6^H_15/2_/^4^F_9/2_→^6^H_15/2_ (LIR2). The comparison with the previously conducted research that employs LIR via energetically higher levels for luminescence thermometry, mentioned above, will be presented at the end of the results section.

## 2. Material and Methods

Metal nitrates (yttrium(III) nitrate hexahydrate, Y(NO_3_)_3_×6H_2_O; dysprosium (III) nitrate pentahydrate, Dy(NO_3_)_3_×5H_2_O; aluminum (III) nitrate nonahydrate, Al(NO_3_)_3_×9H_2_O; Alfa Aesar, purity 99.9%, 99.9%, and 98+%, respectively); citric acid, CA (HOC(COOH)(CH_2_COOH)_2_, Sigma Aldrich, ACS reagent, ≥99.5%); and ethylene glycol, EG (HOCH_2_CH_2_OH, Sigma Aldrich, anhydrous, 99.8%) were used as starting materials without further purification.

In this work, a YAP: 2 mol% Dy^3+^ (Y_0.98_Dy_0.02_AlO_3_) sample was obtained using a modified Pechini method. The desired material was synthesized by adding stoichiometric amounts of metal nitrates to the solution of CA in EG (M: CA: EG = 1: 5: 25), followed by stirring at 80 °C for 30 min, and then at 120 °C until a brownish gel was obtained. The gel was treated at 600 °C for 2 h, annealed at 1100 °C for 2 h, cooled to room temperature, and then ground in a mortar.

The crystal structure of the powder was studied by X-ray diffraction (XRD) using a Rigaku SmartLab diffractometer (Cu-Kα1, 2 radiation, λ = 0.1540 nm) at ambient temperature. The measurements were recorded over a 10 °–90 ° range, with a 0.02 ° step size and 1 °/min counting time. The morphology of the prepared sample was defined by a field emission gun TESCAN MIRA3 scanning electron microscope (SEM). The samples were coated with a thin layer of Au using a typical sputtering technique (Polaron SC502, Fison Instruments, UK)).

The photoluminescence excitation and emission spectra were recorded using a Horiba Jobin Yvon Fluorolog (FL3-22) spectrofluorometer through a fiber-optic bundle using a 450 W xenon lamp as the excitation source; further, the temperature of the sample was regulated by a custom-built hot-stage apparatus [27]. Temperature-dependent emission spectra were then recorded in the 300–850 K temperature range.

## 3. Results & Discussion

### 3.1. Structural and Phase Characterization of YAP: 2 mol% Dy^3+^

**Figure 1:**
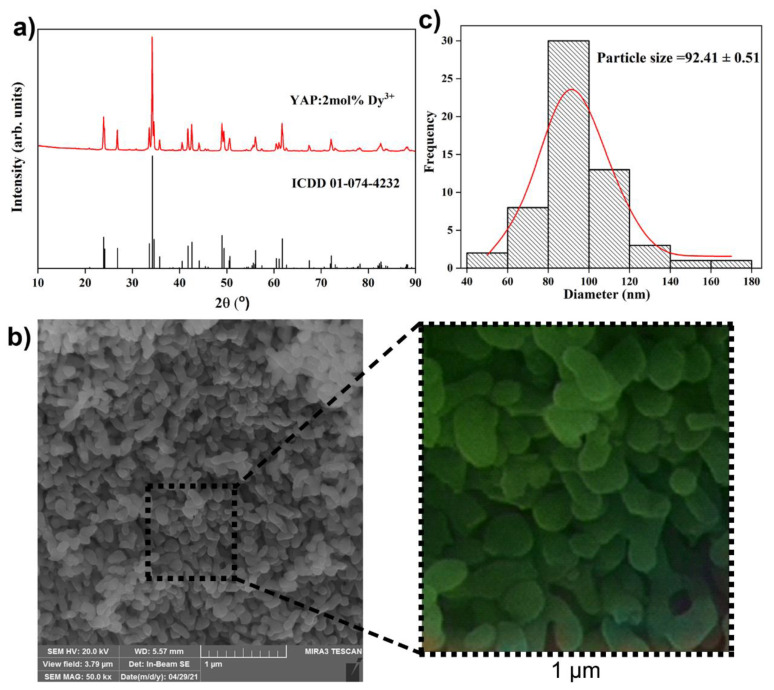
(**a**) X-ray diffraction pattern of the YAP: 2 mol% Dy^3+^ sample. The diffraction peaks are indexed according to the ICDD card No. 01-074-4232; (**b**) scanning electron microscopy images of the YAP:2 mol% Dy^3+^ sample recorder under 50,000× magnification and artificial colorization of 1 μm^2^ by Wolfram Mathematica Neural network; and (**c**) particle size distribution histogram.

X-ray diffraction patterns of the YAP: 2 mol% Dy^3+^ powder sample shown in Figure 1a match the orthorhombic structure with the *Pbnm* (62) space group (ICDD card no. 01-074-4232). No other phases were detected, indicating that the Dy^3+^ dopant was successfully integrated into the YAP host lattice. In YAP, due to their similar ionic radii and balance, Dy^3+^ ions can simply replace Y^3+^ ions (1.027 Å and 1.019 Å, respectively) [28]. The mean crystallite size and appropriate structural parameters were determined using the built-in PDXL2 package software and the initial parameters for the analysis were taken according to the reference [29] (Table 1). The mean crystallite size of the synthesized powder was calculated as ~46 nm.

Scanning electron microscopy was performed in order to explore the powder morphology and micrograph of the sample taken under 50,000× magnification with a corresponding size distribution histogram that is presented in Figure 1b,c. The micrograph shows densely packed, elongated sphere particles the majority of which are between 80 and 100 nm in size (the average particle size is ~92 nm).

### 3.2. Multilevel LIR of YAP: 2 mol% Dy^3+^

**Figure 2:**
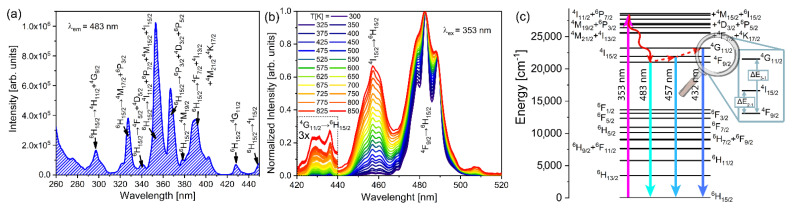
(**a**) Excitation spectrum of YAP:2 mol% Dy^3+^ recorded by monitoring the ^4^F_9/2_→^6^H_15/2_ emission. (**b**) Temperature dependent emission spectra normalized to ^4^F_9/2_→^6^H_15/2_ transition with 420–440 nm spectral range zoomed three times. (**c**) Energy level diagram of Dy^3+^ ions with marked transitions used for LIR1 and LIR2, and the corresponding energy differences ΔE_2-1,_ ΔE_3-1_ (red, dashed upwards arrows represent the thermalization process, straight upward arrow is the excitation, straight downward arrows are emissions, and the red, wavy downward arrow represents the multiphonon de-excitation process).

Figure 2a shows the excitation spectrum of YAP: 2 mol% Dy^3+^ that was obtained by observing the most intense emission attributed to the ^4^F_9/2_→^6^H_15/2_ transition (λ_em_ = 483 nm). Further, this is where the characteristic 4f intra-configurational peaks of Dy^3+^ ions are present at typical positions [30,31,32]: ^6^H_15/2_ → ^4^H_11/2_ + ^4^G_9/2_, ^6^H_15/2_ → ^4^M_17/2_ + ^6^P_3/2_, ^6^H_15/2_ → ^4^F_5/2_ + ^4^D_5/2_, ^6^H_15/2_ → ^4^I_11/2_ + ^6^P_7/2_ + ^4^M_15/2_ + ^4^I_15/2_, ^6^H_15/2_ → ^6^P_3/2_ + ^4^D_3/2_ + ^6^P_5/2_, ^6^H_15/2_ → ^4^M_19/2_, ^6^H_15/2_ → ^4^F_7/2_ + ^4^I_13/2_ + ^4^M_21/2_ + ^4^K_17/2_, ^6^H_15/2_ → ^4^G_11/2_, and ^6^H_15/2_ → ^4^I_15/2_ at ca. 295 nm, 330 nm, 340 nm, 353 nm, 370 nm, 380 nm, 390 nm, 430 nm, and 450 nm, respectively.

Upon excitation at 353 nm, the electrons rapidly de-excite to the ^4^I_15/2_ level via multiphonon relaxation where the large energy difference to the next energetically lower level prevents a further nonradiative de-excitation process. The competing effect to the multiphonon de-excitation process is thermalization, which is process by which the population of higher excited levels are achieved via the thermal energy. The ratio of the optical center population between the thermalized levels is then given by the rates of thermalization and multiphonon relaxation, which is in a steady-state regime summed up as the Boltzmann distribution, as mentioned in the introduction.

Temperature-dependent emission spectra (λ_ex_ = 353 nm), measured in the 300 K to 850 K temperature range and normalized to a ^4^F_9/2_→^6^H_15/2_ transition, are shown in Figure 2b. At longer wavelengths, the emission spectra show Dy^3+ 4^I_15/2_→^6^H_15/2_ (~458 nm, blue) and ^4^F_9/2_→^6^H_15/2_ (~483 nm, blue) transitions, whereas above 600 K, the emission from the third thermalized level, ^4^G_11/2_, arises at shorter wavelengths (~430 nm, violet). In the normalized spectra, both ^4^I_15/2_→^6^H_15/2_ and ^4^G_11/2_→^6^H_15/2_ emission intensities increase with temperature, the latter at a higher rate. Energy level diagrams with indicated emissions and energy differences, ΔE_2-1,_ ΔE_3-1_, are shown in Figure 2c. The levels with efficient populations via thermalization at the investigated temperature range are ^4^I_15/2_ and ^4^G_11/2_ from the ^4^F_9/2_ level.

The traditional LIR1 for Dy^3+^ ions was assessed by the intensity ratio of ^4^I_15/2_→^6^H_15/2_ and ^4^F_9/2_→^6^H_15/2_ transitions, with an energy difference of ΔE_2-1_ (Figure 2c). As shown in reference [18], the emission from the third thermalized level, ^4^G_11/2_, can be used for LIR2 to overcome the limitation of sensitivity obtained by LIR1, as ΔE_3-1_ is substantially larger than ΔE_2-1_.

**Figure 3:**
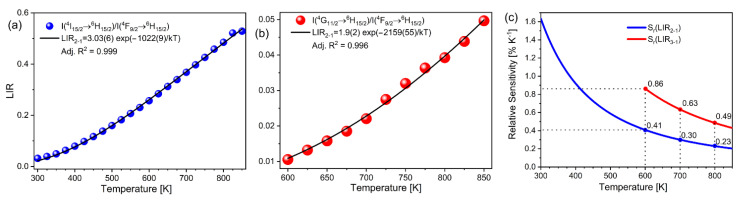
Temperature dependent (**a**) LIR1; (**b**) LIR2 (note: uncertainties are proportional to the point size); and (**c**) S_R_ values obtained by LIR1 and LIR2 temperature readouts.

The fits to Equation (1) are shown in Figure 3a (LIR1) and Figure 3b (LIR2). The high fit quality proves that thermalization is effective and follows a Boltzmann distribution, for both LIR1 and LIR2. As S_R_ (Equation (2)) depends only on the energy difference between levels at a given temperature, the improvement in relative sensitivity for LIR2 in comparison to the traditional, two-thermalized levels LIR1, is linearly proportional to the ratio of the energy difference. The obtained S_R_ values for LIR1 and LIR2 (Figure 3c) decreased with temperature, as Equation (2) predicts, and the relative sensitivity of LIR2 outperforms that of LIR1 by approximately two times at high temperatures. Alas, the approach using the third, higher energy level has its temperature limitations—those at which the emission intensity is not significant enough to be utilized for luminescence thermometry (lower temperature limit) and those at which temperature quenching of the corresponding emissions starts (upper temperature limit). In Dy^3+^- activated YAP, the lower temperature limit for LIR2 was ~600 K where the calculated S_R_ values have the maximum of 0.41% K^−1^ (LIR1) and 0.86% K^−1^ (LIR2) (Figure 3c). Additionally, another limitation is the choice of host materials—temperature quenching should not start before higher excited levels are thermalized; thus, hosts can only be materials that are chemically stable at higher temperatures (such as yttria-stabilized zirconia, vanadates, garnets, silicates, and phosphates) [33,34,35,36]. At 850 K the emission intensity from the highest thermalized level was still increasing. Unfortunately, we could not reach the upper temperature limit for this luminescent thermometer probe due to the limitations of our heating stage.

The comparison of the performance of YAP:Dy^3+^ with other probes that include the thermalization of the 3rd excited level for LIR is given in Table 2. The more extensive comparison of LIR2 and sensitivities for Er^3+^ and Dy^3+^ ions can be found in the literature where the values were obtained theoretically for many hosts from the Judd–Ofelt theory [37,38], while Table 2 only presents and compares the values obtained experimentally. Sr values in Table 2 are given in a format that allows comparisons at all temperatures. The comparison at a single temperature for all the probe materials was not possible as they were investigated at different temperature ranges. Thus, the relative sensitivities are given in the form of (ΔE/k)/T^2^, for the reported values of ΔE in the corresponding literature. As the energy level difference between the 4f levels of lanthanides is approximately host independent, and the relative sensitivity of LIR depends solely on the energy difference between the emitting levels, it is no surprise that the YAP:Dy^3+^ shows similar performance to the other Dy^3+^ investigated probes and also a larger sensitivity than other ions. All three Dy^3+^- activated hosts seen in Table 2, CaWO_4_, YAG, and YAP can be used for high-temperature measurements and assessment of the ultra-high temperature sensing capabilities as well as in a true comparison.

## 4. Conclusions

In this study, three-level luminescence thermometry with Dy^3+^- activated YAP was investigated in order to overcome the sensitivity limitations of Boltzmann thermometers and to obtain a greater relative sensitivity for the LIR readout at high temperatures. As the relative sensitivity at a given temperature is linearly proportional to the ratio of the energy difference, an improvement of approximately two times the relative sensitivity for three-thermalized levels of LIR in comparison to the traditional, two-thermalized levels of LIR is obtained at high temperatures. Three-level thermometry with a higher sensitivity could be used in the following temperature range: beyond temperatures where traditional LIR shows extremely low sensitivity and below the working temperatures of lifetime-based luminescent thermometers. However, the thermometry is limited to the hosts that are chemically stable at high temperatures and to a temperature range in which temperature quenching does not begin prior to the thermalization of higher excited levels. 

## Figures and Tables

**Table 1:** Selected structural parameters of the synthesized YAP:2 mol% Dy^3+^ nanocrystals.

ICDD Card 01-074-4232	YAP:2 mol% Dy^3+^
Crystallite size (nm)	46.2
Strain	0.04
* Rwp	5.88
** Rp	4.27
*** Re	2.81
GOF	2.0906
a (Å)	5.1791
b (Å)	5.3254
c (Å)	7.3694

* Rwp: the weighted profile factor; ** Rp: the profile factor; *** Re: the expected weighted profile factor; and GOF: the goodness of fit.

**Table 2:** Comparison of luminescence thermometry sensor probes that employ the energetically higher thermalized level. The relative sensitivities are given in the form of (ΔE/k)/T^2^. Only the experimentally obtained values were taken into consideration.

Host	Activator	LIR1	LIR2	S_r_ (LIR1)	S_r_ (LIR2)	LIR2 T-Range [K]	Ref.
CaWO_4_	Dy^3+^	^4^I_15/2/_^4^F_9/2_	^4^G_11/2/_^4^F_9/2_	(1664 K)/T^2^	(3473 K)/T^2^	450–800	[18]
YAG	Dy^3+^	^4^I_15/2/_^4^F_9/2_	^4^G_11/2/_^4^F_9/2_	(1500 K)/T^2^	(3545 K)/T^2^	600–938	[19]
NaYF_4_	Nd^3+^	^4^F_5/2/_^4^F_3/2_	^4^F_7/2/_^4^F_3/2_	(1438 K)/T^2^	(2802 K)/T^2^	320–720	[20]
YF_3_	Er^3+^	^2^H_11/2/_^4^S_3/2_	^4^F_7/2/_^4^S_3/2_	(914 K)/T^2^	(1742 K)/T^2^	293–473	[21]
YAB	Gd^3+^	^6^P_5/2/_^6^P_7/2_	^6^P_3/2/_^6^P_7/2_	(728 K)/T^2^	(1611 K)/T^2^	548–873	[22]
**YAP**	**Dy^3+^**	**^4^I_15/2/_^4^F_9/2_**	**^4^G_11/2/_^4^F_9/2_**	**(1470 K)/T^2^**	**(3106 K)/T^2^**	**600–850**	**This work**

## Data Availability

The data presented in this study are available on request from the corresponding author.
